# Stroke health management: Novel strategies for the prevention of recurrent ischemic stroke

**DOI:** 10.3389/fneur.2022.1018794

**Published:** 2022-10-26

**Authors:** Lili Jiang, Yu Zhou, Li Zhang, Lingling Wu, Haibin Shi, Bin He, Yao Wang, Qianghui Liu, Xueli Ji, Xintong Zhang, Lei Jiang, Hao Sun

**Affiliations:** ^1^Department of Emergency, The First Affiliated Hospital of Nanjing Medical University, Nanjing, China; ^2^Department of Interventional Radiography, The First Affiliated Hospital With Nanjing Medical University, Nanjing, China; ^3^Department of Rehabilitation Medicine, The First Affiliated Hospital of Nanjing Medical University, Nanjing, China

**Keywords:** stroke health manager, ischemic stroke, recurrence, health management, follow-up

## Abstract

**Objectives:**

The aim of the study was to assess the effect of the stroke health management model on the prognosis and recurrence of mild to moderate ischemic stroke, guided by the stroke health manager based on the patients' needs. In addition, up-to-date evidence of healthcare resource allocation, planning, and optimization is provided.

**Methods:**

The current research was a retrospective, observational, single-center, history-controlled study with patients divided into two groups, namely, the intervention group and the control group, following the guidance of the stroke health manager. The control group patients received standard medical care during hospitalization, which consisted of advice on healthy lifestyle choices carried out by the bed nurse, but no structured education, WeChat group, or clinical consultation was included. The intervention group patients, in addition to the standard medical care, received health management and health education from the stroke health manager, and after hospital discharge, the patients were followed up over the telephone by the health manager to see if there was any recurrence or readmission.

**Results:**

From 1 January 2018 to 31 December 2020, 382 patients with acute ischemic stroke were enrolled in this study. Through the univariate regression analysis, we found that SHM intervention was associated with a significantly lower risk of recurrence (HR = 0.459). We constructed a nomogram based on the significant variables from the regression analysis and also analyzed the association between the control group and the SHM intervention group among all subgroups using the Cox proportional hazards model to assess the effect of the stroke health management model. Most patients in this study had a total risk point between 170 and 270. The C-index value was 0.76, and the time-dependent AUC for predicting recurrence was >0.7.

**Conclusion:**

The stroke health manager-guided management model based on patients' needs can better control the risk factors of stroke and significantly reduce the recurrence rate of mild to moderate ischemic stroke within 1 year.

## Introduction

In China, the burden of stroke has increased over the past 30 years and has become the leading cause of mortality and morbidity, accounting for 1.57 million deaths in 2018 (1). Notably, ischemic stroke accounts for up to 80% of strokes in China, with a high recurrence rate (1). The Chinese stroke survey showed that the rate of stroke recurrence at 1 year was around 8.2–16% and that the rate of stroke recurrence at 5 years was as high as 41% (2, 3), which is significantly higher than the international report (10–15%) (4).

A study on the risk factors for ischemic stroke in 22 countries (INTERSTROKE) found 10 risk factors that can explain 91.5% of the population-attributable risk of ischemic stroke (4–7). The prevalence of major risk factors for stroke is high in the general population and among stroke survivors, and most of the risk factors have increased over time (1, 5, 8). For preventing recurrent ischemic stroke, the development of measures and standardized strategies targeting etiology and risk factors is important (7, 9, 10). In addition, the intervention of behavioral risk factors for ischemic stroke by improving diet and physical activity, controlling smoking, and limiting alcohol consumption is also of great significance for preventing stroke recurrence (1, 5, 11). Increasing evidence shows that tracking risk factors, clinical characteristics, management patterns, and outcomes of patients with stroke facilitate resource allocation and priority setting in the healthcare system (1, 2, 12–15).

Driven by this principle, the Stroke Screening and Prevention Project Committee of the National Health Commission sponsored the “Stroke Health Manager (SHM)” training program in 2017 for training the stroke health manager nationwide, combined with professional stroke managers and continuous health management (1). The combination of models provides comprehensive, one-stop, and professional stroke health management for patients with stroke (5, 13). A stroke management model should be oriented to the needs of patients with stroke, led by the stroke health manager, and inclusive of multiple disciplines to conduct comprehensive and targeted assessments for patients, through regular and standardized healthcare, medication consultation, rehabilitation guidance, and other stroke management work (9, 15–17).

In this report, a retrospective analysis was conducted to assess the effect of the stroke health management model on the prognosis and recurrence of mild to moderate ischemic stroke, as guided by the stroke health manager based on patients' needs (18, 19).

## Methods

The study design was approved by the Ethics Committee of the First Affiliated Hospital of Nanjing Medical University (Approve ID: 2021-SR-382).

### Study design and participants

This work was a retrospective, observational, single-center, history-controlled study comprising two groups. Based on the electronic medical records, patients who presented to the Stroke Emergency Green Channel of the First Affiliated Hospital of Nanjing Medical University were consecutively recruited from 1 January 2018 to 31 December 2020 by a senior nurse, if they met the following criteria: (1) patients aged ≥ 18 years; (2) patients diagnosed with ischemic stroke as per the *Chinese Guidelines for Diagnosis and Treatment of Acute Ischemic Stroke 2018;* (3) patients with the first episode of ischemic stroke; (4) patients with mild to moderate stroke with an NIHSS score at admission between 1 and 15; (5) patients with a 12-month follow-up after hospital discharge; and/or (6) patients who gave, or authorized a representative to give, informed consent. Patients with incomplete follow-up information were excluded from the study. The included patients were divided into a control group (1 January 2018 to 30 April 2019) and an SHM intervention group (1 May 2018 to 31 December 2020), following the guidance of the stroke health manager.

In the Hospital Quality Monitoring System (HQMS), recurrent stroke diagnosis and comorbidities were identified by the main diagnosis using National Clinical V.2.0 of the International Classification of Diseases, 10th Revision, disease codes. In the Chinese Stroke Center Alliance (CSCA), stroke diagnosis was determined at discharge. Procedures or interventions were identified by the International Classification of Diseases, Ninth Revision, Clinical Modification, Volume 3.

## Health management

### Stroke health manager

Stroke health manager is a new profession promoted by the Stroke Prevention and Treatment Engineering Committee of China for which senior nurses are recommended. The stroke health manager of our hospital conducted full-time management of patients with ischemic stroke during their hospitalization and followed them up after their discharge from the hospital, based on evidence-based medicine, and as a bridge and coordination between medical research and health management.

### Health education

The stroke health manager used multimedia devices to conduct half-hour courses for all hospitalized patients with stroke and their family members in the stroke center. The course content was in line with the American Heart Association/American Stroke Association Guidelines (11), which included management of risk factors for stroke, prevention of deep vein thrombosis in lower extremities, healthy dietary guidance, early exercise rehabilitation therapy, and health education on constipation (13). It was among the stroke health manager's responsibilities to ensure the quality and continuity of the educational content so that patients could learn more about stroke prevention and care during hospitalization as well as make correlative video and promotional films (e.g., ankle pump exercises), which were to be played on the display screen of the outpatient hall and treatment area.

### Clinical consultation

The control group patients received standard medical care during hospitalization, which consisted of advice on healthy lifestyle choices carried out by the bed nurse, but no structured education, WeChat group, or clinical consultation was included. The intervention group patients, in addition to standard medical care, received health management and health education from the stroke health manager, which include the following processes: (1) evaluating patients and establishing health records; (2) adding patients to the group of stroke health management; (3) establishing follow-up relationship and formulating the health education plan; (4) guiding patients to cooperate with doctors for diagnosis and treatment; (5) providing health education through information technology, such as nursing information system, WeChat official account, and health management WeChat group; and (6) conducting full-time “face-to-face” follow-up with patients, carrying out weekly patient education lectures, or hosting irregular in-hospital training meetings and other forms.

### Post-discharge advice

**Figure 1:**
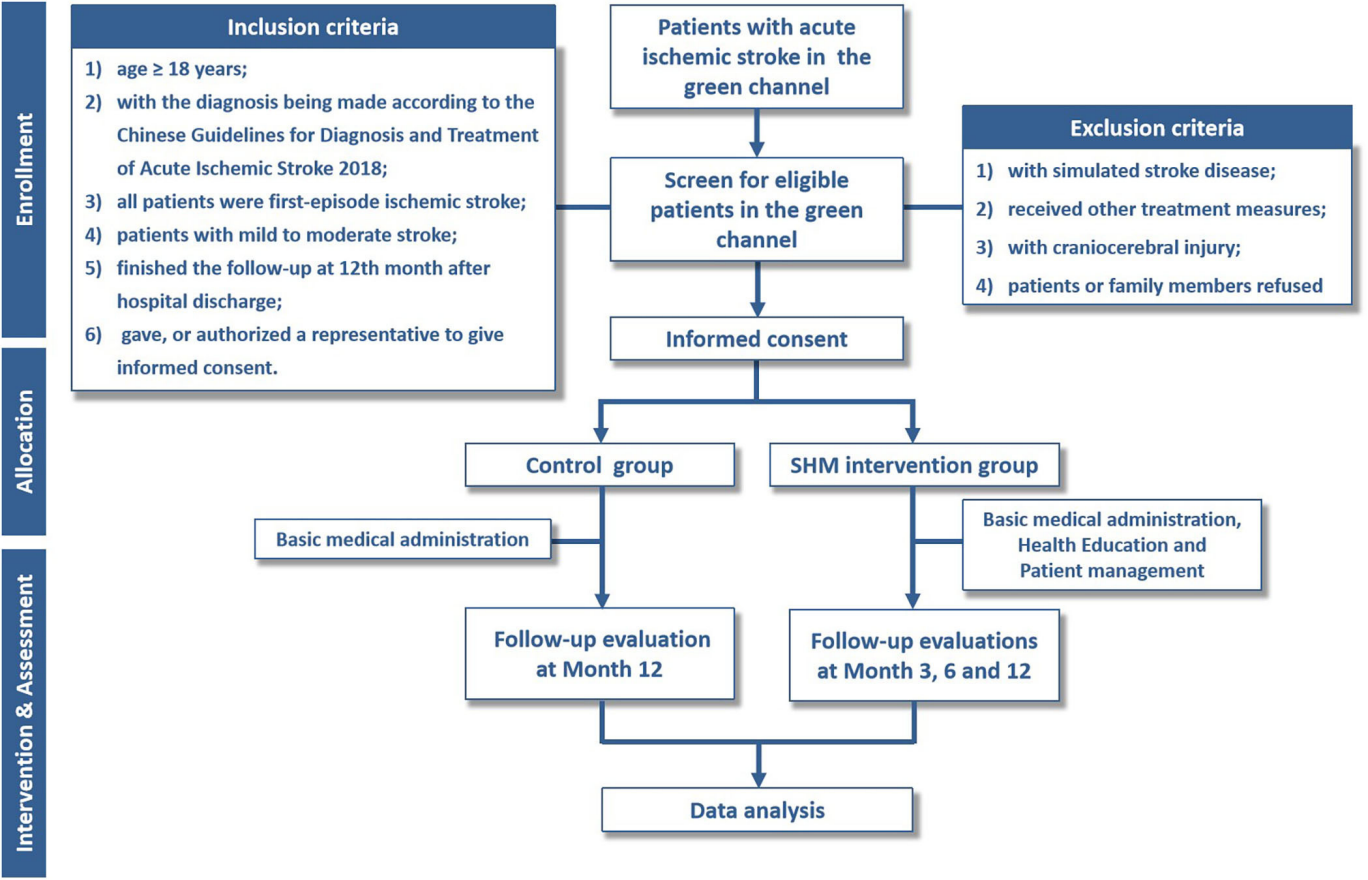
Flowchart of the trial. SHM, stroke health manager.

The stroke health manager issues a follow-up notice to patients who plan to discharge from the hospital, informing them of relevant matters, and a follow-up manual to the patients. The management of patients after hospital discharge is carried out through WeChat public groups, telephone follow-ups, and face-to-face follow-ups. A telephone follow-up is conducted 3, 6, and 12 months after hospital discharge for patients who have not been followed up in the follow-up clinic, including (1) checking the patient's rehabilitation status; (2) guiding healthy lifestyle and chronic disease management; and (3) evaluating the existing or potential risk factors and scale (Figure 1).

### Data collection and outcome assessment

The stroke health manager used a paper-based registry for collecting study data. We used the results of patients' examination the day before their hospital discharge as baseline data, which were retrieved from the medical record. The results of the tests conducted in the follow-up clinic 3 months after discharge were used as outcome indicators, which included sitting systolic blood pressure (SBP), diastolic blood pressure (DBP), serum lipids, modified Rankin Scale (mRS), and recurrence rate. Blood pressure was measured in the seated position after 5 min of rest. An mRS score ≤ 2 at 3 months post-discharge was defined as a good functional outcome. A Barthel index score ≥ 60 was defined as basic self-care in daily life. Fasting blood glucose was not a routine examination for all patients, and only patients with a history of diabetes as listed in the follow-up manual were given a blood glucose examination during the follow-up. Blood pressure and blood glucose levels were compared only between patients with hypertension and diabetes. At 6 months after discharge, the patients were followed up over the telephone by the health manager to see if there was any recurrence or readmission. All the data were then entered into a specialized database.

### Statistical analysis

Descriptive statistics were used to summarize sociodemographic and health-related characteristics. The chi-square test and the *t*-test were conducted to compare the baseline characteristics between the control group and the SHM intervention group. The distribution of variables is given as mean and SD for continuous variables and as number and percentage for categorical variables. For comparison between the two groups, the Mann–Whitney U test was used for continuous variables. All significance tests were two-sided and conducted at a 5% significance level. The recurrence probabilities were estimated using the nomogram. The concordance index (C-index) and the area under the time-dependent receiver operating characteristic curve (time-dependent AUC) calculated by bootstrapping were used to evaluate discriminative ability. Calibration plots were used to evaluate calibrating ability. C-index and AUC values varied from 0.5 to 1.0, where 0.5 represents random chance and 1.0 indicates a perfect fit. However, C-index and AUC values >0.7 suggest a reasonable estimation.

## Results

### Demographic and clinical characteristics

From 1 January 2018 to 31 December 2020, 382 patients with acute ischemic stroke were enrolled in this study (from 1 January 2018 to 30 April 2019, 169 patients in the control group; from 1 May 2019 to 31 December 2020, 213 patients in the SHM intervention group). The characteristics of the study participants are shown in Table 1. The baseline characteristics of the participants included in the analysis were well balanced. In total, 193 (50.66%) participants received intravenous thrombolysis therapy, 99 (25.98%) participants received mechanical thrombectomy, and 90 (23.62%) participants received combination therapy. In the CT scan, 51.57% of the patients showed a unilateral implicative range, with 50.52% of the involved vasculum being basal ganglia. From CTA results, the mainly involved vasculum in 54.71% of the patients was the middle cerebral artery. The most common comorbidities in this population were hypertension (57.59%), diabetes mellitus (23.82%), and atrial fibrillation (20.68%).

**Table 1:** Demographics of the study population.

**Patient characteristics**	**Total (*****n*** = **382)**		**Control (*****n*** = **169)**		**SHM intervention (*****n*** = **213)**		***P***
**Gender**, ***n*** **(%)**							0.350
Men	241	(63.10)	111	(65.68)	130	(76.92)	
Women	141	(36.91)	58	(34.32)	83	(49.11)	
**Age in year, mean (SD)**	67.46	(11.52)	67.23	(11.53)	67.95	(11.55)	0.544
**BMI, mean (SD)**	23.91	(3.00)	23.63	(3.34)	23.93	(2.60)	0.332
**Time to treatment, mean (SD)**	3.1	(2.52)	2.92	(2.68)	3.21	(2.09)	0.241
**NIHSS at admission, mean (SD)**	11.37	(6.27)	11.51	(6.48)	11.37	(6.03)	0.820
**Therapy Interventions**, ***n*** **(%)**							0.052
Intravenous thrombolysis	193	(50.52)	96	(56.80)	97	(57.40)	
Mechanical thrombectomy	99	(25.92)	37	(21.89)	62	(36.69)	
Both	90	(23.56)	36	(21.30)	54	(31.95)	
**Implicative range in CT**, ***n*** **(%)**							0.127
Unilateral	197	(51.57)	83	(49.11)	114	(67.46)	
Bilateral /Multiple	166	(43.46)	70	(41.42)	96	(56.80)	
No abnormality	19	(4.97)	16	(9.47)	3	(1.78)	
**Involved territory in CT**, ***n*** **(%)**							
Basal ganglia	193	(50.52)	81	(47.93)	120	(71.01)	0.102
Lateral ventricle	166	(43.46)	68	(40.24)	98	(57.99)	0.258
Cerebral cortex	126	(32.98)	54	(31.95)	72	(42.6)	0.702
Semioval center	24	(6.28)	12	(7.10)	12	(7.10)	0.557
Brainstem	19	(4.97)	6	(3.55)	13	(7.69)	0.254
Cerebellum	6	(1.57)	4	(2.37)	2	(1.18)	0.412
**Implicative range in CTA**, ***n*** **(%)**							0.626
Unilateral	239	(62.57)	104	(61.54)	135	(79.88)	
Bilateral/Multiple	62	(16.23)	31	(18.34)	31	(18.34)	
No abnormality	74	(19.37)	32	(18.93)	42	(24.85)	
**Mainly involved vasculum in CTA**, ***n*** **(%)**							
Carotid artery	84	(21.99)	35	(20.71)	49	(28.99)	0.591
Vertebral artery/Basilar artery	41	(10.73)	19	(11.24)	22	(13.02)	0.774
Middle cerebral artery	209	(54.71)	84	(49.70)	125	(73.96)	0.080
Anterior cerebral artery	23	(6.02)	9	(5.33)	14	(8.28)	0.611
Posterior cerebral artery	26	(6.81)	11	(6.51)	15	(8.88)	0.837
**Comorbidities**, ***n*** **(%)**							
None	44	(11.52)	23	(13.61)	21	(12.43)	0.254
Hypertension	220	(57.59)	105	(62.13)	113	(66.86)	0.075
Diabetes mellitus	91	(23.82)	45	(26.63)	41	(24.26)	0.086
Atrial fibrillation	79	(20.68)	28	(16.57)	51	(30.18)	0.077
Surgical operation	69	(18.06)	30	(17.75)	39	(23.08)	0.888
Ischemic stroke	62	(16.23)	28	(16.57)	34	(20.12)	0.873
Cardiovascular disorders	36	(9.42)	19	(11.24)	14	(8.28)	0.107
Malignant tumor	19	(4.97)	9	(5.33)	10	(5.92)	0.778
Hyperlipidemia	12	(3.14)	25	(14.79)	40	(23.67)	0.303
Thyropathy	8	(2.09)	3	(1.78)	5	(2.96)	0.698
Valvular heart disease	7	(1.83)	2	(1.18)	5	(2.96)	0.471
Neurological/Psychiatric disorder	7	(1.83)	2	(1.18)	5	(2.96)	0.471
Hemorrhagic stroke	6	(1.57)	3	(1.78)	3	(1.78)	0.775
**NIHSS at discharge, mean (SD)**	5.94	(7.92)	6.55	(9.38)	5.46	(6.53)	0.182
**mRS at discharge, n (%)**							0.617
0	71	(18.59)	28	(16.57)	43	(25.44)	
1	83	(21.73)	38	(22.49)	45	(26.63)	
2	71	(18.59)	33	(19.53)	38	(22.49)	
3	55	(14.40)	26	(15.38)	29	(17.16)	
4	39	(10.21)	19	(11.24)	20	(11.83)	
5	49	(12.83)	11	(6.51)	38	(22.49)	
6	14	(3.66)	14	(8.28)	0	(0)	
**mRS at 3 months**, ***n*** **(%)**							/
0	45	(11.78)	0	(0)	45	(26.63)	
1	47	(12.30)	0	(0)	47	(27.81)	
2	43	(11.26)	0	(0)	43	(25.44)	
3	42	(10.99)	0	(0)	42	(24.85)	
4	18	(4.71)	0	(0)	18	(10.65)	
5	18	(4.71)	0	(0)	18	(10.65)	
6	0	(0)	0	(0)	0	(0)	
**mRS at 6 months**, ***n*** **(%)**							/
0	49	(12.83)	0	(0)	49	(28.99)	
1	48	(12.57)	0	(0)	48	(28.40)	
2	38	(9.95)	0	(0)	38	(22.49)	
3	43	(11.26)	0	(0)	43	(25.44)	
4	19	(4.97)	0	(0)	19	(11.24)	
5	15	(3.93)	0	(0)	15	(8.88)	
6	0	(0)	0	(0)	0	(0)	
**mRS at 12 months**, ***n*** **(%)**							0.122
0	122	(31.94)	62	(36.69)	60	(35.50)	
1	90	(23.56)	44	(26.04)	46	(27.22)	
2	53	(13.87)	18	(10.65)	35	(20.71)	
3	54	(14.14)	14	(8.28)	40	(23.67)	
4	20	(5.24)	5	(2.96)	15	(8.88)	
5	17	(4.45)	4	(2.37)	13	(7.69)	
6	26	(6.81)	22	(13.02)	4	(2.37)	

SHM, stroke health manager; BMI, body mass index; NIHSS, National Institutes of Health Stroke Scale; CT, computed tomography; CTA, computed tomography angiography; mRS, modified Rankin Scale.

### SHM intervention and the risk of recurrent ischemic stroke

**Figure 2:**
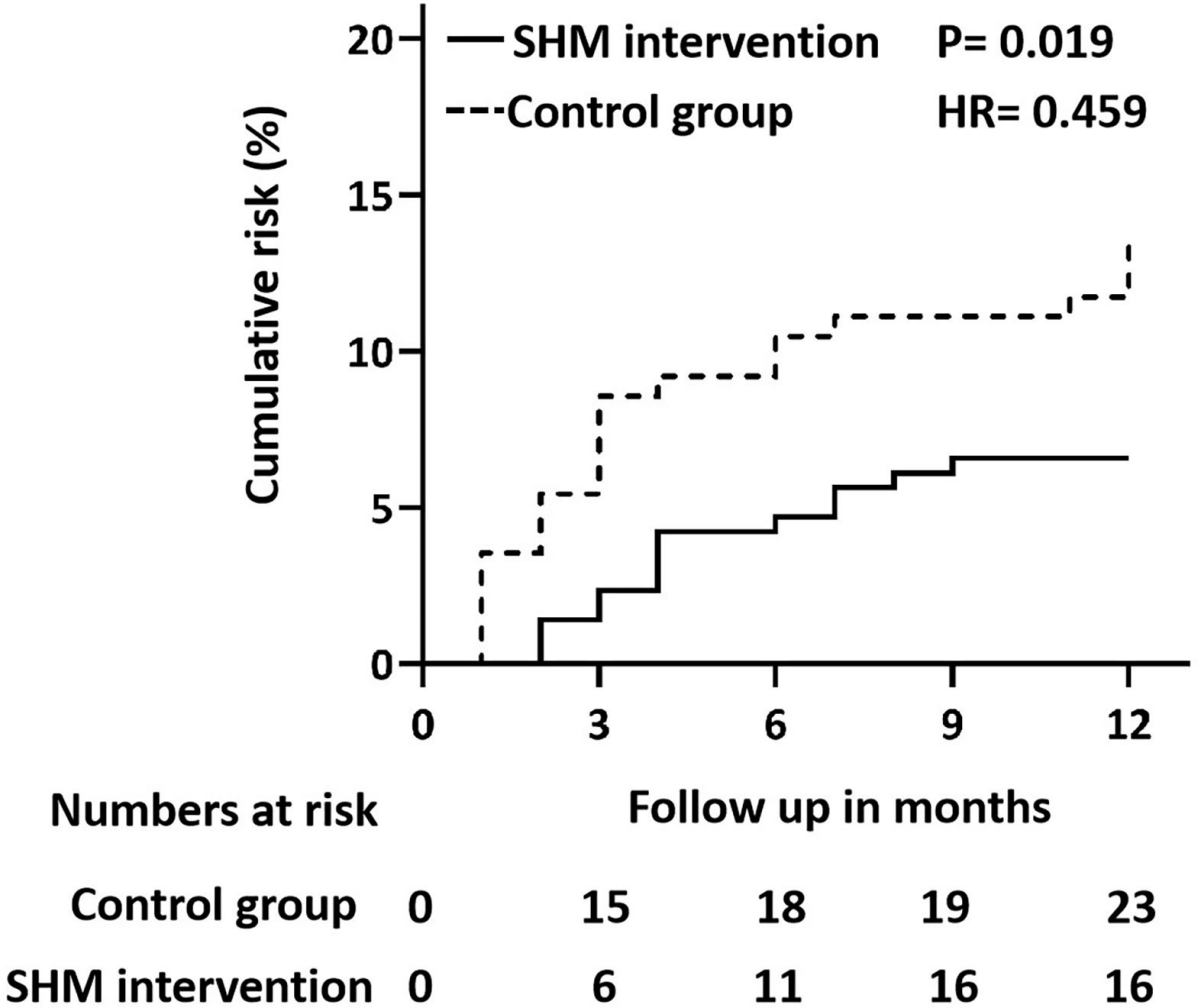
Cox regression model (proportional hazards model) plot for recurrent ischemic stroke in the control group and SHM intervention group. SHM, stroke health manager.

We analyzed the association between the SHM intervention and the risk of ischemic stroke recurrence using the Cox proportional hazard models (Figure 2). The univariate regression analysis revealed that SHM intervention was associated with a lower risk of recurrence (HR = 0.459), showing a 54.1% risk reduction in the SHM intervention group compared with the control group.

### Nomogram variable screening and nomogram construction and validation

The results of the univariate regression analysis are given in Table 2. We found that two variables (SHM intervention and atrial fibrillation) were significantly associated with recurrence. In a multivariate regression analysis of SHM intervention, age, NIHSS on admission, atrial fibrillation, history of ischemic stroke, and treatment modality interventions, three variables (NIHSS on admission, atrial fibrillation, and history of ischemic stroke) were identified as independent prognostic factors for recurrence.

**Table 2:** Univariate and multivariate logistic analyses of variables for the prediction of recurrence rate of ischemic stroke.

**Variable**	**Univariate analysis**		**Multivariate analysis**			
	**OR**	**95% CI**	***P***	**OR**	**95% CI**	***P***
**SHM interventions**	0.470	0.233–0.950	0.035			
**Gender**	1.097	0.542–2.221	0.796			
**Age in year**	1.032	1.000–1.064	0.052			
**BMI**	0.915	0.815–1.027	0.130			
**Time to treatment**	0.926	0.773–1.108	0.400			
**NIHSS at admission**	1.053	0.999–1.110	0.056	1.068	1.013–1.127	0.015
**Therapy interventions**						
Intravenous thrombolysis	1.000					
Mechanical thrombectomy	0.714	0.270–1.885	0.496			
Both	2.038	0.947–4.384	0.069			
**Implicative range in CT**						
No abnormality	1.000					
Unilateral	0.536	0.143–2.018	0.536			
Bilateral/Multiple	0.530	0.138–2.028	0.354			
**Involved territory in CT**						
Basal ganglia	0.614	0.307–1.232	0.170			
Lateral ventricle	0.812	0.402–1.640	0.562			
Cerebral cortex	1.512	0.751–3.045	0.247			
Semioval center	0.401	0.053–3.062	0.378			
Brainstem	1.875	0.519–6.771	0.337			
Cerebellum	1.949	0.221–17.152	0.548			
**Implicative range in CTA**						
No abnormality	1.000					
Unilateral	1.035	0.399–2.682	0.944			
Bilateral/Multiple	2.179	0.744–6.383	0.155			
**Mainly involved vasculum in CTA**						
Carotid artery	0.830	0.374–1.841	0.647			
Vertebral artery/Basilar artery	0.958	0.321–2.860	0.939			
Middle cerebral artery	0.850	0.424–1.704	0.647			
Anterior cerebral artery	1.482	0.418–5.251	0.542			
Posterior cerebral artery	0.543	0.176–1.674	0.288			
**Comorbidities**						
None	1.621	0.634–4.146	0.313			
Hypertension	1.549	0.778–3.084	0.213			
Diabetes mellitus	1.838	0.878–3.848	0.107			
Atrial fibrillation	0.206	0.048–0.875	0.032	0.168	0.039–0.726	0.017
Surgical operation	1.106	0.463–2.639	0.821			
Ischemic stroke	2.175	0.990–4.775	0.053	2.267	1.013–5.072	0.046
Cardiovascular disorders	0.958	0.277–3.308	0.945			
Malignant tumor	1.138	0.252–5.138	0.866			
Hyperlipidemia	0.584	0.199–1.712	0.327			
Thyropathy	1.384	0.165–11.574	0.764			
Valvular heart disease	0.000	0.000–∞	0.999			
Neurological/Psychiatric disorders	1.619	0.189–13.836	0.660			
Hemorrhagic stroke	0.000	0.000–∞	0.999			
**NIHSS at discharge**	1.021	0.984–1.061	0.272			
**mRS at discharge**						
0	1.000					
1	4.196	0.876–20.106	0.073			
2	5.656	1.192–26.828	0.029			
3	1.990	0.321–12.346	0.460			
4	7.547	1.484–38.380	0.015			
5	1.468	0.200–10.791	0.706			
6	9.409	1.409–62.844	0.021			

SHM, stroke health manager; BMI, body mass index; NIHSS, National Institute of Health Stroke Scale; CT, computed tomography; CTA, computed tomography angiography; mRS, modified Rankin Scale.

**Figure 3:**
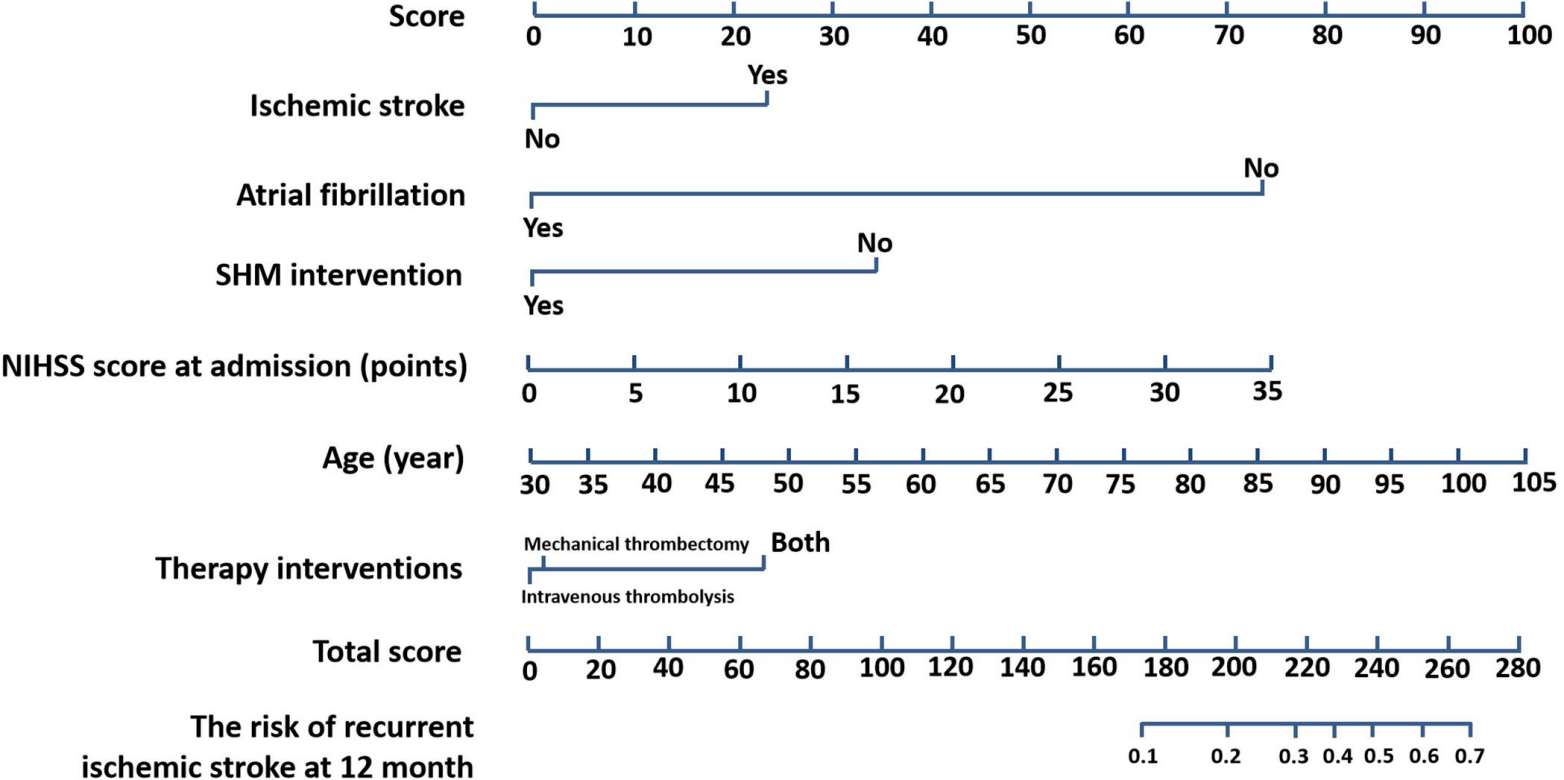
Nomogram for prognostic prediction. Nomogram (SHM intervention, age, NIHSS score at admission, atrial fibrillation, ischemic stroke, therapy interventions) for predicting the risk of recurrent ischemic stroke at 12 months. The importance of each variable was ranked according to the standard deviation along nomogram scales. SHM, stroke health manager; NIHSS, National Institutes of Health Stroke Scale.

We constructed a nomogram based on these identified variables. Figure 3 shows the total score based on the individual scores calculated using the nomogram; most patients in this study had a total risk point between 170 and 270. The C-index value was 0.76, and the time-dependent AUC for predicting recurrence was >0.7, which indicated that the nomogram was beneficial for discrimination.

### Subgroup analyses between the control and the SHM intervention group

**Figure 4:**
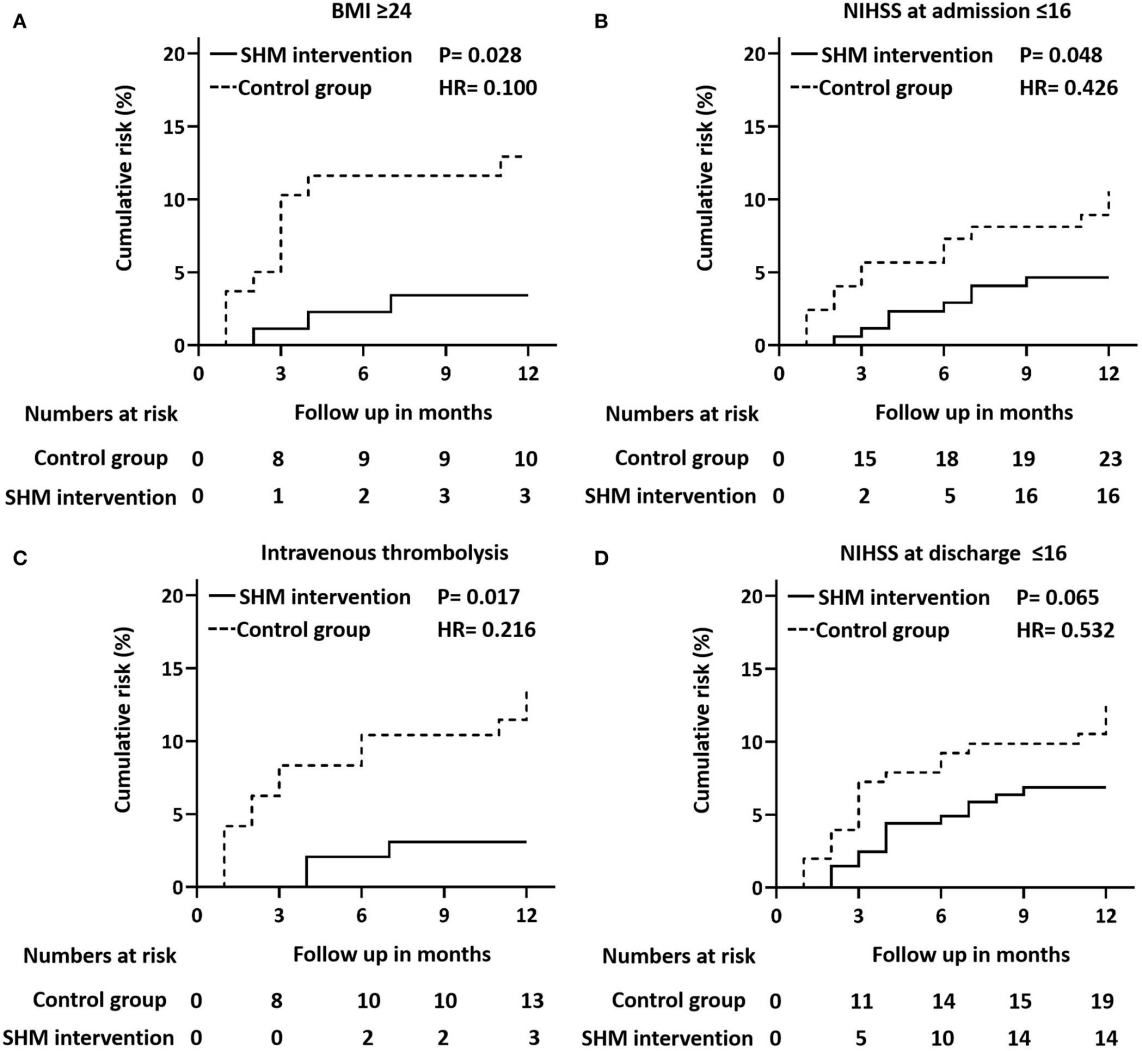
Cox proportional hazard model plots. Cox proportional hazard models plots of outcomes stratified by BMI ≥ 24 kg/m^2^ **(A)**, NIHSS at discharge ≤ 16 **(B)**, intravenous thrombolysis **(C)**, and NIHSS at admission ≤ 16 **(D)**. *P*-values for the overall comparison of the difference between the control group and the SHM intervention group. BMI, body mass index; SHM, stroke health manager; NIHSS, National Institutes of Health Stroke Scale.

We analyzed the association between the control group and the SHM intervention group among all subgroups using the Cox proportional hazards model. Figure 4 shows that the SHM intervention group was associated with a lower risk of recurrence in all four subgroups. In the BMI ≥ 24 kg/m^2^ subgroup, the SHM intervention group had a 90.0% lower risk of recurrence than the control group (*P* = 0.028; HR = 0.459). In the admission at NIHSS ≤ 16 subgroup, the SHM intervention group had a 57.4% lower risk of recurrence than the control group (*P* = 0.048; HR = 0.426). In the intravenous thrombolysis subgroup, the SHM intervention group had a 78.4% lower risk of recurrence than the control group (*P* = 0.017; HR = 0.216). In the discharge at NIHSS ≤ 16 subgroup, the SHM intervention group had a 46.8% lower risk than the control group (*P* = 0.065; HR = 0.532).

## Discussion

Controlling risk factors and continued investment in public health projects have helped reduce the stroke burden in the United States over the past 100 years (16, 20). Conversely, in China, risk factors are highly prevalent among patients with stroke, and the prevalence of major risk factors for stroke in the general population increased from 2002 to 2012 (5). Furthermore, according to the data from NESS-China 2013, the most prevalent risk factors in stroke survivors were hypertension (84.2%), smoking (47.6%), and alcohol use (43.9%) (1, 2). The Chinese government has implemented several public education and primary prevention initiatives for stroke, with some success (2). From 2002 to 2012, the awareness rate, the treatment rate, and the control rate of hypertension improved by 16.3, 16.4, and 7.7%, respectively, although the stroke incidence is still considered at a high level (1, 16). About one-half of patients who survive an ischemic stroke or TIA are at an increased risk of recurrent stroke within a few days or weeks of the initial event, with the greatest risk during the first week (1, 5). Recurrent events lead to prolonged hospitalization, worsened functional outcomes, and increased mortality (12, 16).

In this study, in the intervention group, the stroke health management model oriented to the needs of patients with stroke, and guidance and positive stroke management work was used. During the 1-year follow-up, blood pressure, fasting blood glucose, and LDL-C levels of the patients significantly decreased compared with those before the SHM intervention, that is, the control group. This indicates that the knowledge of stroke prevention and treatment provided for patients and their families should be improved by the stroke health manager, as well as the enthusiasm and initiative of patients for self-management. We also observed the effect of age, NIHSS score, atrial fibrillation, history of previous ischemic stroke, and treatment modalities on recurrence. In this study, the rate of stroke recurrence in patients with a history of atrial fibrillation was reduced in the SHM group (consistent with the China Stroke Statistics 2019) (1). It may be due to the strong awareness of risk factors created by the stroke health manager and high compliance of intervention group patients with atrial fibrillation. Furthermore, it can be concluded from the multivariate logistics regression analysis that ischemic stroke patients with diabetes mellitus should pay more attention to early prevention and early rehabilitation. It may be because the lifestyle change for patients with diabetes is difficult. The full-time follow-up plays a poor role in supervising and controlling underlying diseases.

China is still in its infancy in the whole process of ischemic stroke diagnosis and follow-up process dominated by the stroke health manager, and the work of relevant foreign health consultants has been carried out earlier. The follow-up method is better than the one-way method of sending health education information. In addition, several information methods (e.g., information sent by an AI system and auto-telephone calls) can be used to reduce the manual workload and expand the follow-up population. For example, in 2016, an app called ICTUS3R was developed in Italy specifically to educate patients with stroke by providing information regarding symptoms and coping measures of early stroke, and to encourage people to actively change risky lifestyles; the suitable for all ages part (21) serves as a good inspiration for people to pursue the recommended course.

In future, we should establish health records and track patients' health status for long-range lifetime, improve patient compliance and health awareness, and change lifestyle to promote the control of other chronic diseases in addition to stroke (3, 19). The standardization and humanization of the whole process of stroke health management could improve the knowledge and compliance of patients, which is beneficial to the prognosis of the disease (6, 19, 22). Selection bias is inevitable for a single-center, history-controlled study; therefore, the results should be further verified and validated with larger sample studies.

## Conclusion

The stroke health manager-guided management model based on patients' needs better controlled risk factors for stroke and significantly reduced the recurrence rate of mild to moderate ischemic stroke within 1 year (1, 3–5, 11, 13, 23). This study corroborates the role of the stroke health manager and recommends their use in the secondary prevention of ischemic stroke.

## Data availability statement

The original contributions presented in the study are included in the article/supplementary material, further inquiries can be directed to the corresponding author/s.

## Ethics statement

The studies involving human participants were reviewed and approved by the Ethics Committee of the First Affiliated Hospital of Nanjing Medical University. The patients/participants provided their written informed consent to participate in this study.

## Author contributions

HSu, LeJ, and XZ were responsible for conceptualization, methodology, statistical analysis, and original draft—review and editing. XJ, LiJ, and LZ were responsible for designing the study. LW, HSh, QL, BH, and YW enrolled the participants and collected data. HSu and XJ revised the original manuscript and reanalyzed the data. All authors contributed to the interpretation of the data, critical revision, and approval of the manuscript. All authors read and approved the final manuscript.

## Funding

This work was supported by the National Natural Science Foundation of China (Grant No. 81701872), the Clinical Capability Improvement Project of Jiangsu Province Hospital (Grant JSPH-MC-2021-5), and the Key Clinical Projects of Jiangsu Provincial Finance (Grant (2020)155).

## Conflict of interest

The authors declare that the research was conducted in the absence of any commercial or financial relationships that could be construed as a potential conflict of interest.

## Publisher's note

All claims expressed in this article are solely those of the authors and do not necessarily represent those of their affiliated organizations, or those of the publisher, the editors and the reviewers. Any product that may be evaluated in this article, or claim that may be made by its manufacturer, is not guaranteed or endorsed by the publisher.
